# Standardization for Data Generation and Collection in the Dairy Industry: Addressing Challenges and Charting a Path Forward

**DOI:** 10.3390/ani15020250

**Published:** 2025-01-17

**Authors:** Michel Baldin, Jeffrey M. Bewley, Victor E. Cabrera, Kevin Jones, Connie Loehr, Gustavo Mazon, Juan D. Perez, Matthew Utt, Jeff Weyers

**Affiliations:** 1 Milc Group, San Luis Obispo, CA 93401, USA; mbaldin@milcgroup.com; 2 Holstein Association USA Inc., Brattleboro, VT 05301, USA; jbewley@holstein.com; 3 Department of Animal and Dairy Sciences, University of Wisconsin, Madison, WI 53706, USA; gustavo.mazon@wisc.edu; 4 Ghost Hollow Consulting, Oologah, OK 74053, USA; 5 Summit Farms, Plymouth, WI 53073, USA; connie.loehr@gmail.com; 6 IDEA Solutions, Quebradillas 00678, Puerto Rico; jdperez@ideasolpr.com; 7 Zoetis, Parsippany-Troy Hills, NJ 07054, USA; matthew.utt@zoetis.com; 8 Zinpro Corporation, Eden-Prairie, MN 55344, USA; jweyers@zinpro.com

**Keywords:** data curation, machine learning, data integration, data management, data guidelines

## Abstract

Standards for data generation and collection are important for integration and for achieving data-driven actionable insights in dairy farming. Data integration and analysis are critical for advancing the dairy industry, enabling better decision-making, and improving operational efficiencies. This commentary paper discusses the challenges of and proposes pathways for standardizing data generation and collection based on insights from a multidisciplinary group of stakeholders. Drawing from a series of meetings of industry experts, academics, and farmers organized under the Dairy Brain Project’s Coordinated Innovation Network (CIN), we explore the benefits of creating uniform data generation and collection protocols to ensure compatibility and reliability across different data sources. Key insights include the importance of defining standardization at both farm and industry levels, the role of education and incentives, and the potential for using existing frameworks such as the International Committee for Animal Recording. Additionally, we highlight industry-specific case studies, including successful examples from Brazil such as GERAR, which focuses on reproductive performance data, and Labor Rural, which integrates data from multiple farms to provide valuable insights to farmers and milk processors. The paper concludes with recommendations for implementing these protocols and highlights the need to foster collaboration among stakeholders for the successful implementation and adoption of standardized data generation and collection protocols in the dairy industry.

## Simple Summary

The development of data recording standards is important for the modern dairy industry. This paper summarizes a series of discussions from dairy industry experts. During the meetings, researchers, industry representatives, and farmers discussed current challenges and proposed solutions for establishing data standards in the dairy industry. It was highlighted that both on and off-farm factors make data standardization difficult in the dairy industry. Hence, it was suggested that a collaborative approach between academia, industry, and the government is needed for the successful establishment of data recording standards in the dairy industry.

## 1. Introduction

Dairy farmers and consultants are increasingly reliant on data to make data-driven decisions to improve animal production, health, welfare, and farm economic sustainability. However, the lack of standardized protocols for data generation and collection at the farm level creates significant downstream challenges, not only for farmers and consultants, but for the whole value chain. Effective data integration and analysis are important steps for driving productivity, profitability, traceability, and sustainability in modern dairy farming.

However, variability in data generated by different commercial technologies [1] highlights the need for standardized data generation methods to ensure consistency in data interpretation across farms and contexts. Furthermore, issues with on-farm data generation quality such as inconsistency, inaccuracy, or even missing data can create major bottlenecks regarding the successful implementation of automation and artificial intelligence (AI)-driven tools for data-driven decision-making in dairy farming.

This commentary paper summarizes the discussions from two stakeholder meetings that focused on assessing current challenges and opportunities as well as proposing meaningful actions towards the standardization of data generation and collection within the dairy industry with an emphasis on data standardization at the farm level.

## 2. Materials and Methods

### 2.1. Approach

We utilized a subgroup of the Dairy Brain Coordinated Innovation Network (CIN) [2,3] to address the critical issue of standardization of data generation and collection in dairy farms. The CIN is an initiative led by the University of Wisconsin–Madison to address data management challenges in dairy farming that fosters collaboration among farmers, researchers, industry professionals, and technology developers to enhance data integration, improve decision-making, and promote sustainable practices. Voluntary participants were recruited via dairy-related email listservs to attend two 90-minute meetings to discuss the current practices and challenges regarding data generation and collection in the dairy industry.

A total of 9 people participated in the meetings. Participants included experts from academia (*n* = 2; VEC and GM), the dairy industry (*n* = 6; MB, JB, KJ, JDP, MU, and JW), and one dairy farmer (CL) who shared their vision, insights, and experiences to address the proposed issue. The meetings were conducted by the same facilitators (VEC and GM) via Zoom (Zoom Video Communications Inc., San Jose, CA, USA) in June and July 2024. The primary goal of the group was to ensure that data collected across different platforms and levels are compatible and reliable, thus facilitating its future integration and analysis. More specifically, the group aimed to identify current challenges, propose solutions, and suggest a framework for the standardization of data generation and collection protocols in dairy farms.

Prior to the first online meeting, participants were given the following question to start the discussion: “What do you see as the biggest challenge in achieving standardization in data generation and collection within our industry, and how do you think we can overcome it?”. The meeting followed a semi-structured guide that was designed by the facilitators to allow each participant time to answer the proposed question and to guide the group towards aspects relevant to the group’s main objectives. Meetings were recorded and transcribed by a commercial service (Read AI Inc., Seattle, WA, USA) and made available to all participants at the end of each meeting. Following each meeting, the main discussion and action points discussed were summarized by the facilitators and made available to freely read and edit in a collaborative online document for all participants.

We analyzed the data from the meetings using a qualitative thematic analysis approach. Meeting transcripts were reviewed, and recurring topics were identified and categorized into key themes such as ‘Technological Limitations’, ‘Diverse Data Collection Practices’, and ‘Industry Resistance’. This process involved coding the transcripts to capture both explicit and implicit patterns of discussion. To address differences in opinions among participants, we documented and synthesized contrasting perspectives, ensuring that the paper reflects a balanced representation of the diversity of viewpoints expressed. This analytical process allowed us to draw meaningful insights while maintaining the integrity of the discussions.

### 2.2. Reflectivity Statement

In qualitative studies, especially when interviews are conducted, it is important to acknowledge that the involvement of the authors with the topic may be a potential source of bias on the collection and interpretation of data [4,5]. Hence, it is important to disclose that all discussion participants and authors in the present paper are directly involved with the dairy industry and have more than a decade of experience in their areas. To avoid biases in the discussion, the lead academic (VEC) ensured that the discussion group was composed of academic, industry, and farm representatives. However, we must highlight that, despite the recruiting attempts, the discussion group did not include members of the public, dairy lobbyists, or legislators. Hence, in this paper, we will summarize and explore the challenges and proposed solutions regarding the standardization of data generation and collection that were discussed and summarized by the voluntary participant group previously described.

To mitigate potential bias arising from the dual role of the authors as facilitators and analysts, several measures were implemented. First, meeting transcripts were anonymized during the thematic analysis to reduce the influence of participant identities on interpretation. Second, detailed meeting notes were reviewed collaboratively by multiple authors to cross-check interpretations and ensure objectivity. Additionally, efforts were made during the discussions to foster an inclusive environment where all participants could freely express their views. This included setting clear guidelines for equitable participation at the outset and actively encouraging quieter participants to contribute their perspectives. These measures aimed to minimize bias and ensure that the conclusions drawn reflect a balanced and comprehensive view of the discussions.

One major discussion point was the role of interfaces and the interaction between humans and technology. Because certain data points are generated automatically (e.g., via rumination collars), others require manual entry in data sheets or in herd management software. Ensuring standardization in these processes is complex, especially given the variability in how key data points such as health events are recorded. For instance, the same event might be termed differently across farms or even within the same farm, if different software allow for user-defined naming conventions.

Moreover, there was an emphasis on developing uniform protocols within farms to ensure data consistency over time. This includes having clear documentation and guidelines on data entry practices, which can help new employees maintain consistency with previously established data entry practices. An example discussed was incentivizing employees to ensure accurate data entry by linking it to performance metrics or bonuses, like practices already in place for milk quality or calf care.

Additionally, the group considered the use of advanced technologies such as AI and machine learning to aid in the standardization process. These technologies can help identify patterns and inconsistencies in data, providing a layer of oversight that supports human efforts in maintaining data quality. However, the fundamental principles of standardization, such as having consistent and uniform data entry practices, remain crucial even as these technologies evolve.

## 3. Summary of Discussion

### 3.1. Importance of Standardization

Standardization in data generation and collection is crucial for integrating data from different sources largely because it ensures data quality and compatibility between datasets. Some of the dimensions commonly used to assess the quality of a dataset include the following:

- Completeness: the dataset has no missing records.
- Uniqueness: the dataset does not have duplicated data points.
- Validity: the data points match the required data type and format.
- Timeliness: the refresh rate of the data.
- Accuracy: the correctness of the data values in the dataset.
- Consistency: the data remains uniform over time.
- Fitness for purpose: the data meets the business’ needs.

Out of these seven dimensions, uniqueness, validity, accuracy, and consistency seem to be the ones most affected by lack of standardization in data generation and collection in dairy farms. These dimensions can be undermined even further when data points are created through an unguided and/or unstructured interaction between the user (e.g., a human being) and the technology (e.g., hardware, gadget, or software application). The lack of standard protocols leads to fragmented and inconsistent datasets, which then hampers streamlined and efficient data analysis and consequently hinders progress in dairy farming. By establishing uniform protocols for data generation and collection, the industry can ensure that data collected in different farms, by different personnel, and from different equipment, software, or system, can be seamlessly integrated and analyzed.

### 3.2. Challenges in Achieving Standardization

**Figure 1:**
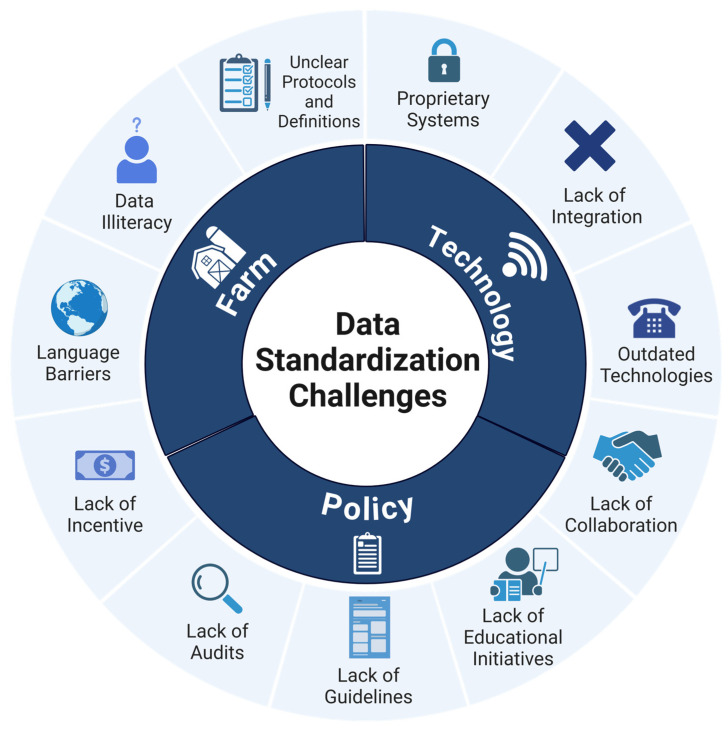
Visual representation of the current data standardization challenges identified by a group of dairy industry experts. The inner layer categorizes the challenges in three main areas. The outer layer details specific challenges observed within each area.

The group identified several challenges that currently complicate the adoption and establishment of standardized data generation and collection methods in the dairy industry. A visual representation of the current challenges raised by the group is shown in Figure 1.

#### 3.2.1. Diverse Data Collection Practices

Dairy farms vary widely in their profile, from small family-run farms to large operations. Small farms may use simple data generation and collection methods or even carry out the manual input of records, which are normally managed by a single or few individuals. Large farms, on the other hand, might employ more advanced software systems that are used by multiple employees. Additionally, different management practices and different educational backgrounds of those collecting and managing data are also observed across farms. Even within a single farm, the use of different biosensors can lead to inconsistent data, further complicating the standardization process [6]. This diversity is one of the factors that leads, for example, to different terminologies and classifications being used to describe the same data point within or between farms [7,8]. In turn, this creates inconsistent and fragmented datasets, posing a significant challenge for data standardization and analysis. Implementing standard protocols that accommodate for this diversity without imposing excessive burdens on operations is a significant challenge for the standardization of the data generation and collection process in the dairy industry. Some farms rely heavily on the implementation of Standard Operating Procedures (SOPs) for everything from feed management to animal treatments. This reliance on SOPs may indicate that such operations could be more inclined to adopt data standardization protocols. Although it may appear that larger farms are more likely to implement SOPs, it was agreed by the group that the inclination towards adopting standardized protocols might be more influenced by ownership profile and management style rather than farm size.

Changing behavior at the farm level is challenging as owners, managers, and farm personnel are accustomed to their existing practices and may resist adopting new standardized protocols. Effective communication and demonstration of the benefits of the implementation of standard data generation and collection protocols are necessary to overcome resistance to change. However, the ownership or management of many dairy farms may not have the resources or be in a position to undertake this task alone. Hence, collaboration and the participation of multiple industry stakeholder categories are likely needed for the effective dissemination and implementation of these strategies. Focusing on how data are recorded at the farm level can help. For example, standardizing variable names within the same farm to ensure consistency over time can significantly improve data quality, even if complete standardization across farms is not immediately feasible. This approach can help farms benchmark their performance across years, even if inter-farm comparisons remain challenging.

For example, the Good Health Records Setup Guide for Dairy Comp 305 (R) Users from the Washington State University, Veterinary Medicine Extension (https://s3.wp.wsu.edu/uploads/sites/2147/2015/04/DC305-SetupGuideweb1.pdf; accessed on 7 November 2024), provides a practical framework for standardizing health event data entry protocols. The guide emphasizes the importance of consistent data entry practices, including specific guidelines for recording disease episodes, treatment details, and other critical health information. By adopting similar structured data entry protocols, dairy farms can improve the consistency and accuracy of their health records, facilitating better data integration and analysis. The document highlights the need to capture all disease episodes accurately, using specific event names consistently, and recording detailed information such as treatment and lesion locations. Implementing such standardized practices can help overcome the challenges posed by diverse data collection methods and improve data quality across different farm sizes and operational styles.

#### 3.2.2. Educational Deficiencies

Thorough employee training during onboarding and continued education are not always common practices in many dairy operations. The lack of standardized training for farm workers involved in data generation and collection directly impacts the quality and uniformity of the generated datasets. Our group highlighted the need for documentation provided by applications or consultants to ensure that data are understood and used correctly by analysts and other stakeholders. Furthermore, we acknowledged the significant challenges associated with the language barrier that may exist between farm workers and farm owners/managers, or with industry personnel helping implement protocols. Therefore, any attempts to provide training or implement standard protocols must focus on delivering content in the language that is best understood by the recipient.

Data literacy also varies significantly among dairy farm owners, managers, workers, and often industry professionals. While some may be adept at using complex hardware and software tools, others may struggle with basic concepts of best practices for data entry. Basic data literacy is paramount when there is an interaction between humans and technology used to create and collect data points. Deficiencies in this area likely explain some of the missing or incorrectly entered data points, inconsistent naming of the same variable over time or across farms, and related factors that result in the creation of irreconcilable datasets. We propose educational programs that aim to address both basic skills and the technical knowledge necessary for effective data generation and collection. These programs should cover foundational concepts, such as accurate data entry and understanding key metrics, as well as advanced technical training on using data analysis tools and integrating hardware/software systems. To ensure program accessibility and effectiveness, we recommend offering certification upon completion, which could serve as a motivating factor for participation and demonstrate competence to stakeholders. Hence, developing tailored training programs to improve data literacy across the industry is essential to ensure future adoption of standard data generation and collection protocols. Leveraging technology for training, such as online modules, webinars, and mobile apps, can help overcome educational disparities and provide accessible, on-demand training.

By incorporating guidelines like management software setups into training programs, farms can ensure that their employees are well-versed in standardized data entry practices. This can include training on the use of consistent abbreviations, capturing detailed treatment and disease information, and maintaining the order of information in records. For example, the guide outlines three simple rules of good recording: capturing all disease episodes, recording episodes using specific events for each disease, and recording the same information in the same order with the same abbreviations for each disease. Adhering to these principles can enhance data literacy and improve the quality and uniformity of health records in dairy farms. Recognizing the diversity in educational backgrounds and language proficiencies among farm workers, training materials should be developed in multiple languages and tailored to different literacy levels, using formats like visual aids, videos, and interactive modules. Programs like Brazil’s ‘Mais Leite Saudável’, which combines technical assistance and training to improve dairy production, provide a model for integrating education with practical applications. Additionally, industries such as healthcare have successfully implemented tiered certification systems, ensuring standardized protocols are taught effectively while offering tangible incentives for participation.

#### 3.2.3. Technological Limitations

The group highlighted that many hardware and software currently adopted in the dairy industry were not designed with the end user in mind. This issue is even more prevalent with legacy technology that primarily expressed the engineer’s vision without too much focus on being user-friendly. For over a decade now, industries such as smartphones and social media apps have been leveraging the power of user interface design to increase user engagement and drive the outcome of the interaction between user and product. A user-friendly interface combined with strategies that guide the user flow positively impact the above-mentioned data quality dimensions that rely on user input. Conversely, product interfaces that are not intuitive, difficult to learn, or fail to guide the user throughout a data collection process, open the opportunity for user error or inconsistency, which is one of the root cause issues of irregularities in datasets. The differences in measurement intervals, duration, and representation among biosensors highlight the technological limitations in standardizing data across various systems [6].

In addition to user interface limitations, many technologies used in our industry may not have the foundational infrastructure needed to implement initiatives that promote data standardization. For instance, legacy products may not support contemporary data formats or have the capabilities to implement global identifier lists that standardize labeling and unique identification of data points across datasets generated by different products. Allowing users to custom-label variables at the farm level is not really the problem; the issue occurs when custom-labeled variables cannot be matched or cross-referenced to a global identification list, which is one of the biggest limiting factors for data analysis across datasets generated by different products. Examples of legacy systems in the dairy industry include standalone herd management software and older milk meter devices that lack compatibility with modern Application Programming Interfaces (APIs) or data-sharing protocols. These systems often require manual data entry or rely on outdated file formats like comma separated values, CSV, which hinders seamless integration with newer technologies. For instance, herd management software launched decades ago may not support dynamic updates or synchronization across platforms, creating significant barriers for farms attempting to adopt standardized data practices.

These systems often have rigid data structures and limited capabilities for data sharing and integration. For instance, older software may not support modern data formats or integration protocols, making it difficult to standardize the data generation and collection processes across different systems. This lack of compatibility across datasets that in theory should contain the same data points hinders efforts to create a unified data ecosystem within the dairy industry. Retrofitting or updating the hardware and software used to create and collect data so that they comply with new standards is both technically challenging and costly. For smaller farms with limited financial resources, upgrading technology can be particularly burdensome. Cost-sharing models, industry partnerships, and government grants such as USDA Rural Development Grants can provide financial support. Collaborative initiatives, where larger cooperatives or companies subsidize technology upgrades for smaller farms, could also help alleviate this challenge. These approaches could ensure equitable access to modern tools and promote the widespread adoption of standardized practices. Technological limitations, including outdated infrastructure and a lack of interoperability between systems, pose significant challenges for current and future data standardization initiatives. Additionally, standardizing software interfaces is unattainable and undesirable because it hampers innovation. Nevertheless, revamping foundational infrastructure such as databases to incorporate concepts like global identification lists, which can be associated with farm-labeled variables, will greatly support efforts to standardize data.

Modernizing legacy systems and developing APIs for data exchange can help alleviate some challenges. Although APIs do not directly solve issues related to the lack of standardization at the source, they can facilitate data standardization downstream and allow for the integration of datasets and the creation of larger databases. Middleware solutions offer another practical avenue for bridging the gap between legacy systems and modern data platforms. By acting as intermediaries, these tools translate and normalize data from older systems, enabling interoperability without the need for complete overhauls. Drawing from successes in the healthcare sector, middleware technologies have been effectively employed to integrate outdated Electronic Health Recording (HER) systems with modern platforms, creating unified networks while preserving historical data integrity. Adopting similar strategies in the dairy industry can significantly accelerate data standardization efforts.

#### 3.2.4. Industry Resistance, Proprietary Systems, and Competition

A significant barrier to standardization is the use of proprietary systems by various companies, which often leads to lack of transparency on how data points are created. These systems are often not designed to integrate with those from other companies, leading to the isolation of the data in data silos. Additionally, companies may be reluctant to share data due to competitive concerns. The group recognized the potential benefits of creating a collaborative environment where companies could share data and information without compromising their competitive edge. This would involve developing industry-wide standards that proprietary systems could align with while maintaining their unique advantages.

Companies often view their proprietary data systems and indexes as a key differentiator that provides a competitive edge. In turn, this may explain why some companies may resist standardization: to maintain a competitive edge by keeping their data proprietary and incompatible with other systems. In the competitive landscape of the dairy industry, data are a valuable asset. For example, a company may adopt different nomenclature to refer to a common variable as an attempt to differentiate themselves in the market. In addition, companies might also use slightly different methodologies or formats to define and calculate indexes that can be used to guide management decisions on the farm. This uniqueness and exclusivity can be misleading and creates challenges for stakeholders that have to generate insights using datasets from different systems.

Companies may be concerned about losing control over their data or exposing sensitive information to competitors. Additionally, companies develop and market multiple sensors, services, and solutions to encompass all sectors of a dairy farm. These “whole farm” solutions offered by a single company might have benefits regarding the standardization of data generation and collection process. Furthermore, in theory, these holistic solutions offered by a single company can be easily integrated, which could facilitate data-driven decisions as it provides farmers and consultants with multiple data sources under a single platform. Despite these possible advantages, these practices create significant barriers to achieving industry-wide data standardization and integration. With the limited data integration possibilities, farmers may become forcefully loyal to a single company as managing data in multiple platforms can be labor intensive and time consuming. As a result, companies may further resist efforts to standardize data generation and collection, fearing that it could diminish their market advantage. Creating incentives for companies to adopt data standardization initiatives, such as grants or market advantages, can encourage adoption and compliance.

Currently, there is limited motivation for companies to adopt standardized protocols, especially if they perceive no direct financial benefit or are not mandated to do so. The adoption of standardized data generation and collection protocols requires a clear value proposition for all stakeholders involved in the dairy industry value chain. Without tangible benefits or incentives, companies may be reluctant to invest in the necessary changes. The absence of regulatory mandates further reduces the motivation to adopt standardized protocols, as companies may prioritize their competitive advantage over industry-wide cooperation to standardize.

### 3.3. Case Studies and Examples

#### 3.3.1. Success Stories in Other Industries

Throughout the discussion, it was evident that standardized data generation and collection guidelines have been successfully developed and established outside of the dairy industry. Hence, it is important to highlight two successful cases of data standardization practices being established and adopted.

In the United States, the healthcare industry has successfully implemented data standardization through government mandates (Title 45 Code of Federal Regulations, Part 170; https://www.ecfr.gov/current/title-45/subtitle-A/subchapter-D/part-170; accessed on 20 November 2024) and industry collaboration. Standardized data formats and HIPPA (the Health Insurance Portability and Accountability Act; https://www.cdc.gov/phlp/php/resources/health-insurance-portability-and-accountability-act-of-1996-hipaa.html; accessed on 25 November 2024) have facilitated data exchange between different healthcare providers, improving patient care and operational efficiency. Lessons from the healthcare industry can be applied to the dairy sector, particularly in developing standardized data formats and incentivizing compliance.

In the agricultural sector, it was highlighted that the European Union has made significant progress in agricultural data standardization through initiatives, mostly from the crop side. These standardization efforts seemed to be driven by crop data integration and analysis initiatives such as publicly available datasets, e.g., Eurocrops (https://www.eurocrops.tum.de/; accessed on 3 December 2024) and the Farm Sustainability Data Network (https://agriculture.ec.europa.eu/data-and-analysis/farm-structures-and-economics/fsdn_en; accessed on 3 December 2024). Thus, these initiatives should serve as an example for the dairy industry to adopt similar approaches to enhance data standardization and integration.

#### 3.3.2. Success Stories Livestock Industry

Despite the lack of widely adopted livestock data generation and collection standards in the United States, our group identified a few noteworthy examples of the power of data standardization protocols in other countries.

In Brazil, a consulting group (Labor Rural; https://laborrural.com/; accessed on 13 January 2025) has successfully integrated data from multiple farms in the agriculture and livestock industry to provide valuable insights to both farmers and companies. This initiative involved standardizing the on-farm data generation and collection practices, regular data quality audits, and close collaboration between consultants, farmers, and companies. The standardized data are used to generate reports and reliable benchmarks to help farmers make data-driven decisions and for companies to tailor their services to meet the needs of a specific farmer or multiple farmers within a region. Under the Educampo project, data from farms—including animal metrics (e.g., lactating cows, production, and reproductive indexes) and economic indicators (e.g., feed costs, veterinary expenses, and opportunity costs)—are collected monthly by uniformly trained consultants using a single software system. Labor Rural’s team conducts rigorous quality control to ensure the accuracy and completeness of the data, which is then used to generate detailed reports. These reports enable farmers to identify inefficiencies and implement targeted interventions, while companies use the insights to tailor services to specific farm needs or regional requirements.

Another Brazilian example is GERAR (Portuguese acronym for Specialized Group in Herd Reproduction; (https://www2.zoetis.com.br/especies/bovinos/gerar/; accessed on 13 January 2025), which focuses on collecting beef and dairy cattle reproductive performance data. This initiative involved veterinarians working on farms, smaller companies, a university, and Zoetis to create a comprehensive value chain of information. They collect, analyze, and report data regarding the performance of different reproduction protocols as well as the performance of different types of semen or bulls. These Brazilian examples, demonstrate the potential of using standardized data to benefit both farmers and companies. Such benefits further highlight the importance of information and standardization in enhancing farm management and industry practices.

GERAR’s success lies in its emphasis on stakeholder collaboration and the use of standardized data protocols to unify reproductive performance data across farms. This approach has improved decision-making by providing actionable insights into reproductive management and protocol performance. Similarly, Labor Rural’s integration of economic and technical farm data into consolidated reports has enabled the identification of bottlenecks and facilitated targeted interventions, leading to measurable improvements in milk production and cost efficiency. For example, farms participating in Labor Rural reported a 10% increase in milk yield attributed to optimized feed strategies informed by the program’s analysis.

In the United States, the Dairy Brain Project (https://DairyBrain.wisc.edu; accessed on 12 January 2025), initiated by the University of Wisconsin–Madison, integrates data from various sources to provide actionable insights for dairy farmers. By developing middleware solutions and collaborating with industry stakeholders, the project has made significant progress in standardizing data collection and integration in dairy farms. The lessons learned from this project can guide future efforts in the dairy industry.

Comparing these initiatives to global programs offers valuable insights into the broader applicability of such efforts. In Europe, the Common Agricultural Policy (CAP) and Farm Accountancy Data Network (FADN) have set benchmarks for integrating farm data through standardized reporting practices. These initiatives emphasize data accuracy and consistency, similar to GERAR and Labor Rural. However, European programs benefit from regulatory support that mandates compliance, whereas GERAR and Labor Rural rely on voluntary participation and incentives. In the U.S., the Dairy Brain Project focuses on leveraging middleware technologies to integrate diverse datasets, aligning with GERAR’s emphasis on creating actionable insights but with a greater focus on advanced analytics. These comparisons underline the importance of adapting strategies to regional contexts while pursuing common goals of data standardization and integration.

Lastly, we must highlight the Europe-based International Committee for Animal Recording (ICAR; https://www.icar.org/; accessed on 15 December 2024) which provides a framework for standardizing animal data. ICAR standards cover various aspects of animal recording, including milk quality, animal identification, and health records. Their standards are widely recognized and used in the livestock industry. However, the dairy industry can yet leverage ICAR standards as a foundation for developing standardized data generation, collection, and annotation protocols. A practical example of this proposition is the current effort between ICAR and the International Dairy Federation (IDF) to develop standards and guidelines for data generated by automated sensors [9]. By adopting these existing frameworks, the industry can streamline the standardization process and ensure consistency across different data sources. The widespread adoption of ICAR standards would also facilitate international data exchange, enabling global benchmarking and collaboration.

### 3.4. Proposed Solutions and Recommendations

**Figure 2:**
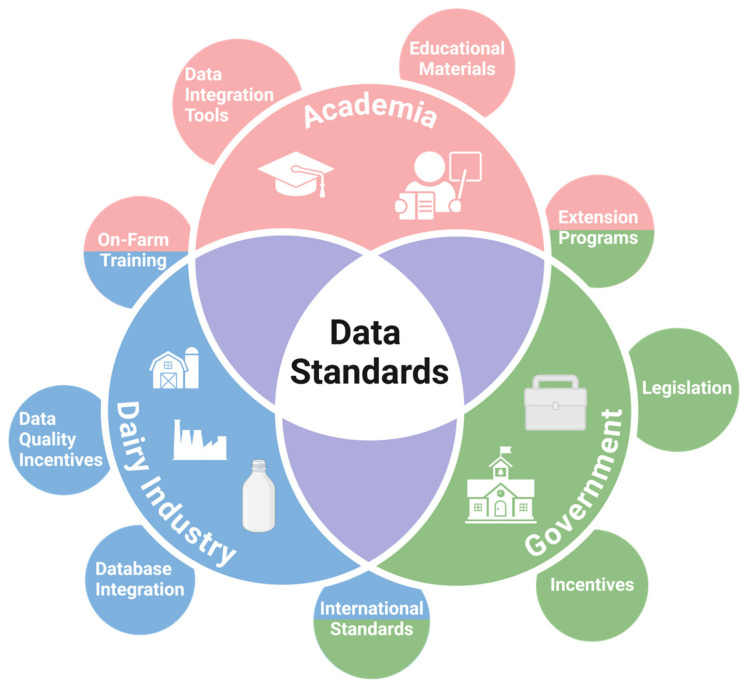
Visual representation of the data standardization solutions proposed by a group of dairy industry experts. The inner layer categorizes the challenges in three main areas. The outer layer details specific challenges observed within each area.

Following the main discussion objectives, the group proposed solutions for the current challenges that are difficult for the adoption and establishment of data generation and collection standards in the dairy industry. Within the dairy industry, the voluntary participants highlighted that the establishment of standardized data generation and collection guidelines should be a conjoint effort involving academics, industry representatives, and the government. A visual representation of the main solutions proposed by the group is shown in Figure 2.

#### 3.4.1. Developing Comprehensive Guidelines

Developing industry-wide guidelines for data generation entry and collection in dairy farms is crucial for achieving successful data integration across the dairy supply chain. The creation of clear and standardized guidelines for data generation and collection at the farm level will ensure data consistency and efficient utilization across different data sources. These guidelines should outline best practices for recording key data points, provide standardized definitions and terminologies, and provide a list of tools and technologies that meet the proposed data standardization guidelines. By ensuring that all farms follow the same guidelines, the industry can create a consistent and reliable dataset that supports analysis and decision-making at the farm level and industry-wide. The costs associated with implementing these guidelines can vary widely. Small farms may face initial expenses ranging from USD 5000 to USD 10,000 for basic upgrades, whereas larger operations could incur costs exceeding USD 50,000 for comprehensive overhauls, including training, software updates, and integration tools. Regional variations further influence these expenses, with more developed markets often requiring higher initial investment but achieving quicker returns due to economies of scale. The guidelines should cover the following:

- Standardized data formats, abbreviations, codes, and protocols.
- Clear definitions of commonly recorded data points.
- Guidance for manually inputted and automatically generated data points.
- Flexibility to accommodate different farm sizes and diverse operational practices, ensuring inclusivity without imposing excessive burdens on any segment.
- A proposition for the dissemination and implementation of the data standardization initiative.

#### 3.4.2. Adopting Existing Frameworks

The dairy industry should leverage existing standards, such as those provided by ICAR, to jumpstart the standardization process. Adopting established frameworks like ICAR standards can provide a strong foundation for farm- and software-based data standardization efforts. These standards offer a comprehensive set of guidelines for recording and managing animal data, which can be adapted to meet the specific needs of the dairy industry. By using these frameworks, the industry can avoid the pitfalls of developing new standards from scratch and ensure compatibility with data systems on a global scale.

Implementing pilot programs to test and refine standardization protocols can help identify challenges and opportunities. These programs can provide valuable feedback and demonstrate the benefits and possible unforeseen bottlenecks of implementing standardized data generation and collection practices. Cost-sharing models can mitigate financial constraints, particularly for small farms. Cooperative initiatives, like those under Brazil’s ‘Labor Rural’, demonstrate how shared infrastructure and data services can reduce costs while ensuring quality. Similarly, government subsidies or incentive programs, akin to the European Union’s CAP, can fund pilot programs to refine and scale standardization efforts. Therefore, the implementation and fine tuning of pilot data standardization programs will be crucial for the successful implementation of such programs at large scale.

Standardization is an ongoing process that requires continuous improvement. Regular reviews and updates to the guidelines, based on feedback and technological advancements, can ensure that the standards remain relevant and effective throughout time.

#### 3.4.3. Enhancing Industry Education and Awareness

Engaging all industry stakeholders in the standardization process is critical for success. Providing training, resources, and incentives can help all participants adopt standardized data practices and take full advantage of their benefits.

Improving data literacy across the whole industry and providing training on standardized data generation and collection practices is expected to significantly enhance data quality. Education and training across all stakeholder categories are critical components for successful data standardization in the dairy industry. Education and training should begin with players directly involved with data creation and collection. Special attention should be given to processes where data are created through an interaction between human and hardware or software, or where data points are manually inputted. Training programs should focus on teaching best practices for data entry, explaining the importance of data standardization, and providing practical tools for consistent data collection. In addition, these programs should be tailored to accommodate differences in language and education backgrounds so that they effectively engage and serve all players in the industry.

Training programs and awareness campaigns can help dairy farm owners and managers, as well as industry professionals, understand the benefits of data standardization. Industry professionals who interact with farms regularly can play a significant role in disseminating best practices. In fact, many of these industry players are part of professional associations that require their members to pursue and report continued education opportunities to maintain their professional memberships. Therefore, professional associations may be an avenue to require or encourage their members to pursue training and continued education related to data standardization.

Furthermore, using technology to provide training and support to industry stakeholders can help reduce educational disparities and time availability constraints. Online training modules, webinars, and mobile apps can be an accessible and effective form of on-demand training, especially for farm employees. Also, remote support tools can help troubleshoot data collection issues in real-time.

Additionally, industry organizations and academic institutions can play a vital role in developing and delivering training programs. By partnering with these organizations, farms can access high-quality training resources and receive help implementing strategies that improve data literacy and promote standardization within their organization.

#### 3.4.4. Leveraging Government and Industry Incentives

Government incentives and industry collaboration are crucial. For example, the Brazilian “Programa Mais Leite Saudável” (PMLS) offers a valuable example of a government-led initiative aimed at improving milk quality and dairy farm productivity through incentivized standardization practices. The program allows dairy cooperatives and industries to utilize presumed credits from PIS/Pasep and COFINS taxes, which can be used for federal tax compensation or reimbursement. As a condition for participating in the program and accessing these financial benefits, the entities must implement projects that promote the development of their dairy producers. These projects typically focus on providing technical assistance, enhancing genetic improvements, and promoting sanitary education in livestock management. Since its inception, the PMLS has benefited over 80,000 dairy producers across more than 2300 municipalities, resulting in significant improvements in milk quality and farmer profitability. The program’s success underscores the importance of combining financial incentives with mandatory project implementation, thus fostering better practices and higher standards within the dairy industry. The PMLS could serve as a model for similar initiatives in other regions, demonstrating how strategic government policies can drive industry-wide improvements and support the adoption of standardized data generation and collection protocols.

To further support adoption, examples from other sectors can be illustrative. For instance, the U.S. healthcare sector’s transition to EHR systems leveraged federal subsidies to offset costs for providers. Similarly, grants for upgrading equipment or public–private partnerships in telecommunications have shown how strategic financial support can drive technological change while benefiting all stakeholders. Adopting similar models for the dairy industry could accelerate standardization adoption, particularly among smaller farms that are more cost-sensitive.

Incentives for standardization can take various forms, including financial subsidies, tax breaks, or market incentives. For instance, companies that comply with data standards or achieve certification for data standardization could receive preferential treatment in government contracts or be eligible for grants that offset the costs of implementation. By making it financially viable for companies to adopt standardized protocols, incentive programs can accelerate the transition to a more integrated and standardized data ecosystem.

These incentives could also include funding for research and development, grants for upgrading equipment, and creating regulatory frameworks that promote data standardization.

#### 3.4.5. Encouraging Multidisciplinary Collaborative Efforts

Building partnerships between industry players, including software developers, equipment manufacturers, and service providers, can promote data standardization. Collaborative initiatives, such as developing interoperable systems and sharing best practices can drive progress. Middleware solutions present a cost-effective alternative to complete overhauls by enabling legacy systems to interface with modern platforms. These solutions can be implemented incrementally, reducing the immediate financial burden on farms. Partnerships between academia, industry, and governments could further subsidize middleware development, as demonstrated by the Dairy Brain project’s collaborative approach. Furthermore, there is an opportunity to involve stakeholders from other industries which have participated in large data standardization and integration initiatives. Bringing collaborators from outside the dairy industry may accelerate the data standardization process as they can provide valuable insights learned from other data standardization initiatives.

Encouraging collaboration between the private sector, universities, government bodies, and representatives of the dairy farmers can foster a more cooperative approach to data standardization. Thus, creating a working group focused on promoting data standardization in the dairy industry can drive standardization efforts. This group can develop guidelines, promote best practices, and coordinate collaborative initiatives.

One proposition is creating platforms for ongoing dialogue and cooperation among stakeholders that can help address emerging issues and refine data standardization practices. Industry professionals, such as veterinarians, nutritionists, and consultants, who frequently visit farms, can play a vital role in disseminating data standardization initiatives. Additionally, these industry professionals can quickly detect pitfalls and roadblocks and help tweak and improve data standardization efforts. These partnerships can leverage the expertise and resources of different stakeholders to address the technical and logistical challenges that usually hamper the dissemination, implementation, and sustainability of new protocols or strategies.

For instance, industry consortia can play a pivotal role in developing common data standards and promoting their adoption across the sector. Academic institutions can conduct research and provide technical support, while industry bodies can advocate for policies and incentives that support standardization efforts. Through collaboration, the dairy industry can create a unified approach to data standardization that benefits all stakeholders. Data standardization guidelines from these collaborative efforts would probably be easier to implement and more beneficial to all stakeholders than mandatory guidelines established by the government.

Collaborative initiatives can include joint research projects, industry consortia, and public–private partnerships that promote data standardization and integration. Through these efforts, the industry can create a unified approach to data management that benefits all stakeholders.

#### 3.4.6. Focusing on the Farm Level and Creating Middleware Solutions

At the farm level, data points are created and collected through one of these two pathways: manually entered by a user; or automatically generated by a technology (hardware or software). Regarding data standardization efforts, despite some overlap between the two methods, each one of these pathways requires a particular set of guidelines and approaches. Some of the primary factors that help ensure data standardization for manually inputted data points are as follows: (I) thorough initial training and continued education of personnel inputting the data; (II) implementation and enforcement of SOPs for data entry; (III) the utilization of software with a user-friendly interface that drives user behavior; (IV) the implementation of software that prioritizes the selection of, rather than free-typing, user-defined approaches; (V) if a software allows the user to custom-name a variable, then, in the database, the custom name must be linked with a global identifier; and (VI) software that prevents user error or at least alerts the user when a user error occurs and allows for the revision of incorrectly entered data.

On the other hand, some of the relevant factors that promote data standardization for automatically generated data include the following:

- In its database, the technology stores measurements, qualifications, processes, or units of information according to pre-established industry standards.
- When data are stored in divergence with industry standards, appropriate documentation should be provided so that data formatting and conversions can be performed by data integrators.
- When an interaction between humans and technology is required, the technology should be capable of identifying and accounting for user error.
- The technology should maintain data consistency and validity over time.

Focusing on standardizing data generated and collected at the farm level is a critical step. However, as discussed above, some approaches must focus on the user (e.g., generally a farm employee), whereas other factors are intrinsic to the hardware or software creating and storing the data. Establishing protocols for data entry, equipment configuration and utilization, and record-keeping can ensure high-quality farm-level data. Developing guidelines that are both comprehensive and flexible enough to accommodate different farm sizes and operational practices is essential. These guidelines should include the following:

- Standard definitions for recording and describing key data points (e.g., milk yield, health events).
- Best practices for data entry and monitoring of automatically generated data.
- Framework to help develop farm-level advocates that can help with the governance of data quality.

In parallel, middleware solutions such as the Dairy Brain initiative can bridge the gap between different data systems, ensuring data interoperability. These solutions can translate and standardize data from various sources, facilitating integration. Developing collaborative platforms and middleware solutions can facilitate data integration from different sources. These platforms can provide a standardized interface for data collection and sharing, translating proprietary formats into a common standard. Middleware solutions can also handle data cleaning and normalization, ensuring that data from different sources can be seamlessly integrated.

#### 3.4.7. Leveraging Advanced Technologies

Advanced technologies, such as IoT (Internet of Things) devices, AI, ML (machine learning), and data analytics platforms can enhance the data generation, collection, and analysis process. In addition, the development of AI, ML models, and data analytics platforms tailored to dairy farming can optimize operations and provide meaningful insights. However, it is important to highlight the efficacy and applicability of these advanced technologies that can be negatively affected by non-standardized and unreliable data. Furthermore, ensuring these technologies are accessible and user-friendly is crucial for their widespread adoption.

IoT devices can significantly enhance data collection by providing real-time, automated data capture. For example, sensors can monitor environmental conditions in barns, track animal movement, and measure feed intake. These devices can transmit data to a central system, reducing the need for manual data entry and improving the accuracy and efficiency of the data collection process. Furthermore, AI and ML can be used to analyze large datasets identifying patterns and insights that may not be apparent through traditional analysis. For example, these techniques can predict health issues in animals based on historical data and current conditions [10,11], allowing for early intervention. These data analysis techniques could be further explored to standardize retroactive data that was collected without standardization methods in place as well as actively standardize data from multiple sources as they are integrated into a single database. Recently, a probability-based standardization method, which converts sensor measurements into cumulative probabilities, has shown promise in integrating non-standardized data from various biosensors [6]. Similar approaches could be further adapted and explored to standardize data collected across different dairy farms and systems.

Automated Milking Systems (AMSs), widely adopted in modern dairy farms, exemplify the integration of advanced technologies that promote standardized data generation. An AMS can capture a wealth of real-time data, including milk yield, milking time, and cow activity, which provides an excellent foundation for ML applications aimed at disease detection and prediction. Recent studies demonstrate how AMS-collected data, such as milk yield, conductivity, and flow, can be utilized in predictive models to detect and manage clinical conditions like mastitis. For instance, one study explored the use of AMS data in multilayer perceptron models to detect changes in milk homogeneity indicative of clinical mastitis [12]. Similarly, another study highlighted the potential of ensemble machine learning techniques to enhance disease prediction accuracy in dairy herds [13]. Additionally, a third study extended these applications by demonstrating advanced predictive capabilities using time-series AMS data for early detection of metabolic disorders [14]. These examples underscore the role of AMS in driving advancements in both data generation and the application of artificial intelligence in dairy farming.

The development of advanced data analytics platforms that integrate data from various sources can provide valuable insights for farm management [2,3]. These platforms can offer dashboards and reports that highlight key performance indicators (KPIs), trends, and areas for improvement. By providing actionable insights, these platforms can help farmers make data-driven decisions highlighting the need for data standards. We believe that, as farmers, industry, and consumers realize the power and utility of these integrated platforms, there could be industry-wide initiatives to incentivize data standardization and facilitate the integration of data into these advanced data analytics platforms.

Other emerging technologies such as large language models (LLMs) can enhance data integration and analysis. These technologies could help bridge gaps between disparate datasets by learning and recognizing patterns across different data sources [15,16]. For example, LLMs could be used to decode and standardize data (e.g., a health event) that is recorded in different ways within and between farms, into a universal code. However, the successful implementation of these models relies on a minimal level of consistency and accuracy of the data recorded, reinforcing the need for robust standardization practices from the outset.

## 4. Conclusions

Standardization in data generation and collection is pivotal for the advancement of the dairy industry. However, farm-related factors such as size, management style, and farmer data literacy pose challenges for the standardization of data. While challenges exist, they can be addressed through targeted education, incentives, and collaborative efforts. By adopting existing frameworks and fostering industry-wide cooperation, the dairy industry can significantly enhance its data capabilities, ultimately leading to more efficient and sustainable farming practices. Addressing these challenges requires a collaborative approach, comprehensive guidelines, and leveraging advanced technologies. By fostering transparency and cooperation, the dairy industry can harness the power of standardized data to drive innovation and sustainability.

## Data Availability

Not applicable. This commentary paper does not involve the generation or use of any datasets. It is based solely on the opinions, expertise, and discussions of the authors.
